# Chiral Derivatives of Xanthones with Antimicrobial Activity

**DOI:** 10.3390/molecules24020314

**Published:** 2019-01-16

**Authors:** Joana Araújo, Carla Fernandes, Madalena Pinto, Maria Elizabeth Tiritan

**Affiliations:** 1 Laboratory of Organic and Pharmaceutical Chemistry, Department of Chemical Sciences, Faculty of Pharmacy, University of Porto, Rua de Jorge Viterbo Ferreira, 228, 4050-313 Porto, Portugal; up201703236@ff.up.pt (J.A.); cfernandes@ff.up.pt (C.F.); madalena@ff.up.pt (M.P.); 2 Interdisciplinary Center of Marine and Environmental Research (CIIMAR), University of Porto, Edificio do Terminal de Cruzeiros do Porto de Leixões, Av. General Norton de Matos s/n, 4050-208 Matosinhos, Portugal; 3 Institute of Research and Advanced Training in Health Sciences and Technologies, Cooperativa de Ensino Superior Politécnico e Universitário (CESPU), Rua Central de Gandra, 1317, 4585-116 Gandra PRD, Portugal

**Keywords:** antimicrobial, xanthones, chirality, chiral derivatives of xanthones, caged xanthones

## Abstract

According to the World Health Organization, the exacerbated use of antibiotics worldwide is increasing multi-resistant infections, especially in the last decade. Xanthones are a class of compounds receiving great interest in drug discovery and development that can be found as natural products or obtained by synthesis. Many derivatives of xanthones are chiral and associated with relevant biological activities, including antimicrobial. The aim of this review is to compile information about chiral derivatives of xanthones from natural sources and their synthesized examples with antimicrobial activity.

## 1. Introduction

According to the Center for Disease Control and Prevention, almost half of all antibiotics prescribed in outpatient clinics are unnecessary [1,2], where the overuse of antibiotics is one of the causes of increasing bacterial resistance [3]. Additionally, the unregulated availability of antibiotics in a community frequently leads to ill-advised self-medication. For example, in certain countries of Africa and Asia, the use of non-prescription antimicrobials is quite frequent, which leads to unnecessary and inadequate consumption, dose, and treatment periods [3]. These behaviors prompt microorganism adaptation rather than treating infections [4], pointing towards an alarming increase of infections triggered by resistant strains. Therefore, treatments tend to be more expensive and with lower efficiency. Infections caused by strains with no response to antibiotics, such as vancomycin-resistant *Enterococcus* (VRE) and methicillin-resistant *Staphylococcus aureaus* (MRSA) are becoming more frequent and fatal [1]. Consequently, research for new antimicrobial agents to fight these pathogens remains a challenge [1]. Frequently, the marked antibiotics interfere with bacterial biosynthesis, which is easily mutated, leading to a loss of activity and development of new resistant strains [5]. Therefore, it is important to develop new antimicrobial agents using different strategies to minimize mutations or other mechanisms of resistance [5].

**Figure 1:**
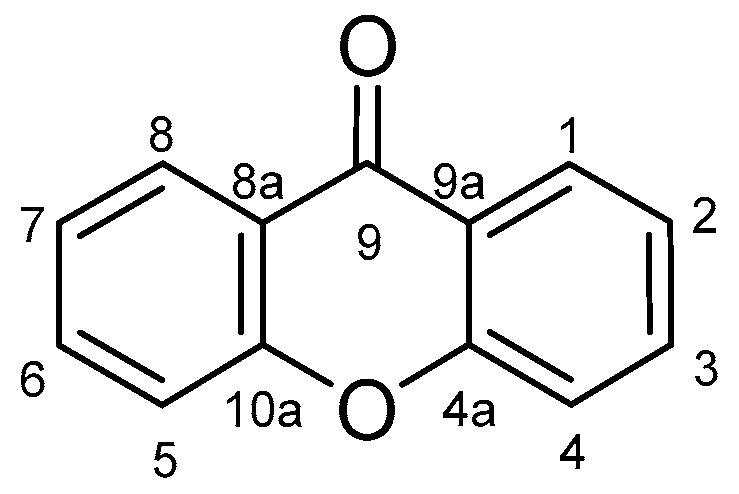
Xanthone scaffold.

Xanthones (9*H*-xanthen-9-one) comprise a family of *O*-heterocycle symmetrical compounds with a dibenzo-γ-pyrone scaffold (Figure 1). The interest of this structure in drug development comes from the wide range of different substitutions that can generate a diverse library of compounds able to modulate several biological responses, and as such, is a privileged structure for drug development [6,7,8].

According to their structures, xanthone derivatives can exhibit a variety of different activities such as antioxidant [9,10,11], vasorelaxant [12], anti-ulcer [13], anti-inflammatory [14], antiallergic [15], cytotoxic [16,17], antimicrobial [8,18,19,20,21], antiviral [17,22], antiplatelets [23], antiarrhythmic and antihypertensive [24], anesthetic [25], among others [8,26,27,28,29]. Their large spectrum of biological activities leads researchers all over the globe to isolate and/or synthesize new xanthone derivatives for medicinal research purposes [30,31,32]. Xanthone derivatives can be isolated from fungi, lichen, higher plants, and other organisms and/or sources from terrestrial and marine environments [33,34,35], or obtained by synthesis [8,29,36]. Among the natural and synthetic xanthone derivatives, many examples present a chiral moiety and enantioselectivity in the biological response.

This review reunites the natural and synthetic chiral derivatives of xanthones (CDXs) with relevant antimicrobial activities. The described configuration of the stereogenic centers, the specific rotation, the enantiomeric ratio, and the enantioselectivity are presented in accordance to the source of the work.

## 2. Natural Chiral Derivatives of Xanthones

Natural products usually are complex structures with multiple stereogenic centers and a wide spectrum of biological activities [26,37,38]. The bulk of the plant extracts with pharmacological activity was established due to their traditional health care use in tribes and indigenous population [9,39,40,41]. Natural xanthone derivatives offer a wide range of biological activities with established pharmacological purposes [42]. One of the most studied xanthones found in nature is α-mangostin, isolated from tropical fruits of *Garcinia mangostana*. These fruits have been used for many decades in folk medicine to treat diarrhea, skin infections, and chronic wounds in Southeast Asia [10,43]. Several studies have been reported about its anticancer and antimicrobial activities, among others [10,14,16,27,43,44,45,46]. The xanthone α-mangostin is not chiral, but many chiral derivatives were isolated and presented interesting antimicrobial activity along with other similar structures.

In order to verify the structure–activity relationship (SAR) of natural CDXs with common chemical groups, such as furan, pyran, hydroxy side chains, and others, the CDXs and antimicrobial activity were reunited in different topics.

### 2.1. Natural CDXs with Furan Groups

Furan derivatives can be found in natural products or synthesized, being associated to a wide range of biological and pharmacological activities [47]. Several natural CDXs with furan groups were isolated and few of them presented antimicrobial activities (Table 1).

Mangostanin (**1N**) was isolated by Nilar et al. [48] and studied by Suksamrarn et al. [46]. Fukai et al. [49,50] focused on *Cudrania cochinchinensis* and *C. fruticosa* and isolated compounds **2N**, **3N**, and **4N**. Boonsri et al. [51] explored the roots of *Cratoxylum formosum* to obtain formoxanthone-C (**5N**).

According to Table 1, all the referred structures presented interesting antimicrobial activity. Toxyloxanthone-C (**2N**) and formoxanthone-C (**5N**) displayed strong activity against fungi and Gram-positive bacteria [21,49,51,52], while formoxanthone-C (**5N**) was also active against Gram-negative bacteria (*S. typhi*) [51]. The configuration of the stereogenic center was described only for formoxanthone-C [51].

### 2.2. Natural CDXs with Pyran Groups

Many pyran derivative compounds with biological properties can be found in nature [53]. Few authors have been exploring their antimicrobial activity among other pharmacological properties [54]. In many natural structures, the xanthone scaffold is merged with pyran group that contains a stereogenic center (*). CDXs with pyran groups were isolated from many different species and displayed antimicrobial activities (Table 2).

Suksamrarn et al. [46] isolated mangostanol (**6N**) and tovophyllin-B (**7N**) from *G. mangostana*. Dharmaratne et al. [55] isolated calozeyloxanthone (**8N**) from *Calophyllum monii* and *C. lankensis*, while smeathxanthone B (**9N**) was isolated from *G. smeathmannii* by Komguem et al. [41]. Namdaung et al. [56] and Makmur et al. [57] investigated artoindonesianin-C (**10N**), found in *Artocarpus rigidus*, and Siridechakorn et al. [53] studied cowagarcinone D (**11N**) in *G. Cowa*.

Calozeyloxanthone (**8N**) revealed an interesting activity against many strains of MRSA and MSSA [55], and tovophyllin-B (**7N**) and artoindonesianin-C (**10N**) presented activity against mycobacterial strain [46,56] (Table 2). Regarding the structural similarity, these compounds (**7N**, **8N**, and **10N**), unlike the others of this group, contain two cycle units that contribute toward increasing the lipophilicity, which is a determinant factor to improve antimicrobial activity [46].

The specific rotations of the compounds smeathxanthone B (**9N**), ${\left[\alpha \right]}_{D}^{22}$ +30.3° (*c* 0.02 MeOH), and artoindonesianin-C (**10N**), ${\left[\alpha \right]}_{D}^{24}$ 0° (*c* 0.16, CHCl_3_), were reported [41,57].

### 2.3. Natural CDXs with Hydroxy Side Chains

Oxygenated and prenylated xanthones have been investigated as new drugs due to their pharmacological properties [58], such as antimalarial [59] and antimicrobial activities [60], among others. Besides these xanthones, only a few structures are found in nature containing hydroxy group in the lateral chains, and some of them displayed interesting antimicrobial activities (Table 3).

Fuscaxanthone I (**12N**) was isolated from *G. fusca* and presented anti-*H. pylori* activity [61]. Caledol (**13N**) and dicaledol (**14N**) were isolated from *C. caledonicum*, and both presented antifungal activity against *A. fumigates* [62]. Antimycobacterial activity was exhibited by mangostenol (**15N**), isolated from *G. Mangostana,* which was evaluated against *M. tuberculosis* [45,46].

The specific rotation was reported only for fuscaxanthone-I (**12N**) and mangostenol (**15N**): $\left[\alpha_{D}^{26}\right]$ −9.5° (*c* 0.20, CH_3_COCH_3_) and $\left[\alpha_{D}^{31}\right]$ −20° (*c* 0.10, MeOH), respectively [61,63]. Neither absolute nor relative configurations were reported.

### 2.4. Natural Caged Xanthones

Another important type of CDXs are the caged xanthones, where one of the aromatic rings of the xanthone scaffold lost the aromaticity to form a bicyclic ring resulting in multiple stereogenic centers.

Caged xanthones are a class of compounds known by their uses in traditional medicine and strong antimicrobial activity [64,65,66], among others [17,67,68]. However, these xanthone derivatives are more often investigated as antitumor agents than antimicrobial due to their potent cytotoxicity activity against various cultured mammalian cancer and drug-resistant cell lines at low concentrations [69,70,71,72,73,74].

A few caged xanthones with antimicrobial activity were reported (Table 4).

Rukachaisirikul et al. [65,75] described the scortechinone structures (**16**–**31N**) and Reutrakul et al. [17,64] reported the prenylated caged xanthones (**32**–**36N**). The specific rotations were measured and the configuration of the stereogenic centers were defined for all of the scortechinones structures (**16**–**31N**) [65,66,75,76] (Table 4). According to the antimicrobial assays, scortechinones B (**17N**) and C (**18N**) stand out due to their promising antibacterial activity against MRSA [75]. It is important to highlight that some compounds are epimers of each other, as for example scortechinone L (**27N**) and scortechinone A (**16N**) in carbon C-15, being the activity of L (**27N**) higher than the activity of A (**16N**), with MIC values of >64 and 128 µg/mL, respectively [65]. This result emphasizes the relevance of the stereochemistry in the development of new antimicrobial agents.

According to Table 4, prenylated caged xanthones (**32**–**36N**) showed little or no activity against MRSA and MSSA strains [17,64].

Additionally, Sukpondma et al. [66] found out that the crude methanol extract from the fruits of *Garcinia hanburyi* was significantly active against MRSA. This discovery led to exploring the antimicrobial activity of the compounds **37**–**41N** present in this extract. These compounds embody a pyran group, which leads to an increase of their activity. Reutrakul et al. [17,64] also reported the antimicrobial properties of some caged xanthones with pyran group (**42**–**44N**) (Table 5).

Comparing the structures and activities from compounds **37N** to **44N** (Table 5), the moreollic acid (**40N**) and morellic acid (**41N**) presented higher activity than the others [64,66]. This suggested that antimicrobial activity comes from the simultaneous presence of a carboxylic group in the prenylated chain in C-8 (according to xanthone scaffold, Figure 1) and another prenyl chain (C-1) [66]. The same conclusion was found by Chaiyakunvat et al. [64] who reported that morelic acid (**41N**) and gambogic acid (**44N**) revealed the greatest activities. Only a few examples measured the specific rotations.

The stereochemistry of the natural caged xanthones is represented in all the structures but their absolute configuration was only described and determined by Ren et al. [71,77] for structures **41** and **44N**, gambogic and morellic acid, respectively.

### 2.5. Other Natural CDXs

Antimicrobial activity of natural CDXs such as kielcorins or structures with glycoside and peptide groups, were also reported. In this subsection, natural CDXs with diverse chemical nature are presented (Table 6).

Coqueiro et al. [78] explored the benefits of *Kielmeyera variabilis*, a tree used in folk medicine to treat several tropical diseases, which is known to harbor active compounds against MRSA, such as kielcorin (**45N**). Another example is mangiferin (**46N**), which comprises a glycoside structure and its pharmacological and biological benefits have been studied for many years [7,79]. In USA, mangiferin can be found in Vimang^®^, an antioxidant commercialized aqueous extract of *M. indica* and *G. mangostana* commonly known to improve human health [7,80]. In addition, mangiferin has been tested as an antiviral treatment [81,82].

Recent studies concern pharmacological properties of mangiferin, such as antipyretic [80] and antimicrobial [79] properties, leading Sigh et al. [79] to explore other derivatives (Table 6). The promising results led the group to develop mangiferin analogues with antimicrobial activity [79,80], which are described in Section 3.2 (Mangiferin Analogues).

In another study, Siler et al. [83] analyzed extracts of *Centaurium* species with antibacterial agents for food preservation. According to this report, mangiferin (**46N**) was considered a good hit structure in antimicrobial drug development [83].

Moon et al.’s studies [84] in *Streptomyces* strains resulted in the discovery of a new secondary metabolite, buanmycin (**47N**), a pentacyclic xanthone with one stereogenic center determined as (*S*)-enantiomer. The antimicrobial potential of these marine strains was explored against *S. aureus*, *B. subtilis*, and *K. rhizophila* (Table 6).

Microluside A (**48N**) is a glycosylated disubstituted xanthone. It was isolated by Eltamany et al. [85] from the broth culture of *Micrococcus sp*. EG45, a species presented in the Red Sea sponge: *Spheciospongia vagabunda* (Table 6).

Wang et al. [86] isolated the first dimer xanthone derivative from the bark of *G. mangostana*, garmoxanthone (**49N**), which announced the strong activity against two strains of MRSA (Table 6).

## 3. Synthetic CDXs

Synthetic derivatives are especially important structures, not only for performing SAR studies, but also to develop new compounds, to increase the chemical diversity, and to increase the biological activities. The majority of synthetic CDXs are inspired in natural xanthone derivatives, to take advantage of their already reported biological properties, and to attempt to improve their biological response [7,31,87].

Despite the fact that natural compounds possess pharmacological applications, their structures are limited to their production, and sometimes, comprise high levels of complexity, making them difficult to extract and purify, and even harder to synthesize. SAR studies are meant to determine the important moieties of natural compounds in order to improve their pharmacological/biological properties with smaller and simple molecules [88,89,90].

The synthesis of small molecules is, normally, an easier procedure being less time-consuming than the processes of extraction, purification, and identification, as well as being economically viable. Additionally, synthesis on a gram scale can be easier to achieve than isolation from natural sources [36,89,90]. Besides, the enantioselectivity in biological assays can be explored because both enantiomers can be obtained via enantioselective synthesis or racemic approach, with further separation of the enantiomers [29,89,91,92].

Throughout this section, the synthetic CDXs, as well as their antimicrobial activity, were compiled according to their structures.

### 3.1. Muchimangins Analogues

Muchimangins are benzophenone-xanthone hybrid polyketides isolated from the roots of *Securidaca longepedunculata*, and are used in traditional Congolese medicine [93]. Among these structures, muchimangin B has been known to induce an apoptotic-like cell death in human pancreatic cancer cells [94]. Kodama et al. [93] synthesized five new muchimangins analogues to develop new antimicrobial agents (Table 7). The compounds presented inhibitory activity against *S. aureus* and *B. Subtilis* [93].

According to the results displayed in Table 7, the enantioselectivity of antimicrobial activity was explored for compounds **1**–**3S,** being the racemate and both enantiomers evaluated against *S. aureus* and *B. subtilis*. Enantioselectivity was evident in compound **3S**, with the dextro enantiomer being more active against *S. aureus* than the levo enantiomer and the racemate. Compounds **4S** and **5S** were assayed as racemates which haven’t displayed any activity against these strains [93].

The SAR studies suggested that the presence of a hydroxy group at C-6 was important for the growth inhibitory activity against both strains, *S. aureus* and *B. subtilis*. Besides that, these results exposed the importance of enantioselectivity studies for the development of antimicrobial agents [93].

### 3.2. Mangiferin Analogues

Singh et al. [79], inspired by the large range of pharmacological activities of mangiferin (**45N**), synthesized new mangiferin analogues (**6**–**11S**) and screened their antimicrobial activity (Table 8) [79].

According to antimicrobial results, mangiferin (**45N**) and analogues revealed powerful activity in the growth inhibition of *S. virchow* and significant antibacterial activity against *B. pumilus* and *B. cereus*. On the other hand, all tested compounds revealed poor growth inhibition of *P. aeruginosa* and low antifungal activity [79].

### 3.3. Amino Acid Xanthone Derivatives

Inspired by natural xanthone properties, and by Dahiya and collaborators [95] work of iodoquinazolinones and nitroimidazoles conjugated with amino acids which presented strong antimicrobial activity, led Chen et al. [96] to synthesize xanthone derivatives with conjugated l-amino acids (Table 9).

According to Table 9, the compounds with the best antimicrobial activity were the ones that were conjugated with l-phenylalanine (**16S** and **26S**), l-tyrosine (**17S** and **27S**), and l-tryptophan (**18S** and **28S**), followed by compounds conjugated with l-cysteine (**19S** and **29S**), l-methionine (**20S** and **30S**), and l-proline (**21S** and **31S**). These compounds contain amino acids with high aromaticity and hydrophobicity, which makes them stable amphiphilic structures. The antimicrobial effect comes from the penetration of the amino acid hydrophobic chains in the bacterial membranes where the cationic moiety of the amino acids interacts with the membrane phospholipids disturbing the bacterial membrane. This is a strategy to develop new antimicrobial agents [96]. Due to the membrane’s essential properties, its disruption would lead to death without mutations resulting in loss of recognition by the antibiotics, leading to ineffective treatments [5].

### 3.4. α-Mangostin Analogues

Cationic antimicrobial peptides (CAMPs) are amphipathic structures with hydrophobic and cationic groups that represent an effective component of the innate immune system against multiple microbes. These structures act by burring the hydrophobic moiety in the membranes core, while the cationic residues disrupt bacterial membrane [5,87,97,98]. Due to the manufacturing costs and poor stability of peptides, Koh et al. [99] developed small molecules with CAMPs essential moieties (**32**–**38S**) (Table 10). The adopted strategy was to use the α-mangostin, a xanthone core with isoprenyl groups, and conjugate the lipophilic side chains with basic amino acids. The aims of the work were to confirm the penetration of the lipophilic chains to enhance the membrane permeability and to examine the role of the cationic moieties by conjugating with basic amino acids (Table 10) [99].

The same strategy was used to develop new anti-tuberculosis agents (**39**–**44S**), which led them to assay a few of the previous compounds (**33S**, **34S**, and **36S**) as antimycobacterial agents (Table 10) [97].

In these studies, α-mangostin was conjugated with l-lysine (**32S**), l-histidine (**33S**), and l-arginine (**34**–**38S**), being **38S** double conjugated with l-arginine [99]. From the compiled results, the structures **34S**, **36S**, and **38S** were the most promising due to their excellent antimicrobial activity, which inspired further evaluation of compounds **34S** and **38S** in more strains of MSSA, MRSA, VRE, and others (Table 10) [99]. These compounds revealed strong activity against Gram-positive bacteria- and multidrug-resistant strains [99]. More recently, Koh et al. [100] tested these compounds in a panel of Gram-negative pathogens: ten strains of *P. aeruginosa*, three strains of *E. coli*, and three strains of *K. pneumoniae* (Table 10) [100].

According to the results, the small size might facilitate the penetration of the external bacterial membrane, where the lipophilic chains in the form of isoprenyl enhance the penetration of the bulky xanthone into the cytoplasmic membrane, and the cationic moiety to form an amphiphilic structure to interact with microbe’s membrane, where the more dispersed the positive charge is, the more disruption and selectivity occurs [99].

Nevertheless, in mycobacterial assays, the compounds **42S** and **43S** revealed potent antimycobacterial activity, which leads to a new class of antimycobacterial agents with hitherto unprecedented modes of action [97].

### 3.5. Xanthone Derivatives with 2-Hydro-3-Amino and Piperazine Groups

Piperazine is a six-member heterocyclic with a broad spectrum of biological activities, which leads research groups to develop new piperazine derivatives [101,102,103]. Besides these, piperazine derivatives are reported as having antidepressant [104], anticancer [105], antimalarial [106] and diverse antimicrobial activities [101,107], among others [108].

Chimenti et al. [109] reported strong anti-*H. pylori* activity of synthesized analogues of *N*-substituted of 2-oxo-2*H*-1-benzopyran-3-carboxamides. Due to similar structural features of these analogues with xanthone scaffold, Klesiewicz et al. [110] synthesized xanthone derivatives with potential anti-*H. pylori* (Table 11). Regarding Klesiewicz et al.’s report [110], the compilation of the results of the antimicrobial assays is described in Table 11.

According to Table 11, the SAR analysis showed that the presence of two hydroxy groups in the amine moiety led to a decrease of activity. This suggested that the activity of the compounds was not only determined by the hydrophilic character but also by the structure and spherical conformation determined by the side chains [110]. Neither configuration of the stereogenic centers nor specific rotations were reported.

### 3.6. Derivatives of Caged Xanthones

In order to carry on the studies of caged xanthones, Chaiyakunvat et al. [64] synthesized some compounds (**64**–**75S)** inspired by the natural structures with antimicrobial activity previously reported (**Table 12**). First, they synthesized compound **75S** that corresponds to the methylated morellic acid (**36N**) (with MIC of 25 µg/mL against MRSA strains). Then, they synthesized morrelic acid derivatives (**64**–**75S**) comprising amino acid moieties, Table 12.

As reported in Table 12, the morellic acid derivatives with more inhibition bacterial growth were the ones with amino acids containing hydrophobic side chain (**64S**, **65S**, **69S**, **71S**, and **72S**) [64]. This state is in agreement with the previous report where the antimicrobial activity was higher in the structures with the hydrophobic and/or aromatic amino acids [64,99]. The configuration of stereogenic centers are presented but specific rotations and absolute configuration were not reported.

### 3.7. Xanthone Derivatives of C-2-Substituted

Szkaradek et al. [18,111] developed interesting studies about antimycobacterial activity using xanthones. They started by the development of new 2-xanthone derivatives with structural moieties with well-known antimycotic properties such as the allyl (**76S**) and morpholine (**77S**) groups [18] (Table 13). Then, synthesized xanthone derivatives C2-substituted to generate new anti-tuberculosis agents (**78**–**88S**) [111] (Table 13).

Szkaradek et al. [18,111] considered that the activity increased with the enlarged size of the lateral chain, due to the mycobacterial membrane containing lipids, which makes the hydrophobic side chains easier to penetrate. According to Table 13, compound **86S** possessed the most promising activity [111]. In this work, the stereochemistry was also ignored.

## 4. Conclusions and Future Perspectives

Among many of natural CDXs, a few compounds where highlighted due to their interesting antimicrobial activity. Mangostanin (**1N**), toxyloxanthone C (**2N**), formoxanthone-C (**5N**), scortechinone B (**17N**), and scortechinone I (**24N**) displayed strong activity against fungus and Gram-positive bacteria, with formoxanthone-C (**5N**) also being active against Gram-negative bacteria. Geronthoxanthones G and A (**3** and **4N**) also presented interesting activities and should be explored along with SAR studies in order to synthesize new analogues.

The synthetic CDXs were inspired by natural scaffolds with potential antimicrobial activity. The most promising strategy among the synthesized CDXs analogues was the development of membrane-targeting potent antibacterial agents in which the lipophilic side chains contain cationic amino acid residues that can penetrate the microbial membranes in order to disrupt them.

Regarding the stereochemistry and enantioselectivity, the configuration of the stereogenic centers are often ignored and only a few examples described the antimicrobial activity for both enantiomers and/or racemate. Differences in the activity among enantiomers or epimers were observed. One example concerns the naturally occurring epimers of scortechinone A (**16N**) and L (**27N**), with **27N** being more active. Another interesting example concerning the different activities of racemic or pure enantiomeric forms are the synthesized muchimangins **1S** and **3S**.

It was found that the use of l-amino acids in the majority of the synthesized analogues amplified the interaction with the antimicrobial membrane for a major effect. These examples emphasize the importance of chirality in the development of new antibiotics.

## Figures and Tables

**Table 1:** Antimicrobial activity of natural CDXs with furan groups.

No.	Name/Structure	Antimicrobial Activity (MIC)
**1N**	Mangostanin 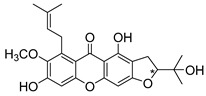	*Mycobacterium tuberculosis* H_37_Ra (25 µM)
**2N**	Toxyloxanthone-C 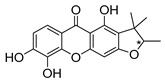	*Candida albicans* (25 µM); *Candida glabrata* (8 µM); *Aspergillus fumigatus* (8 µM); *Aspergilus nidulans* (8 µM); *Cryptococcus neoformans* (8 µM); *Bacillus substilis* PCI-219 (3.13 µM); MSSA JMC-2874 (6.25 µM); MRSA (6.25 µM); *Micrococcus Luteus* (12.5 µM)
**3N**	Gerontoxanthone-G 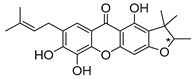	*B. subtilis* PCI-219 (12.5 µM); MSSA JMC-2874 (12.5 µM); MRSA (12.5 µM); *M. luteus* (12.5 µM)
**4N**	Gerontoxanthone-A 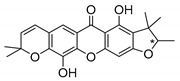	*B. subtilis* PCI-219 (<25 µM); MSSA JMC-2874 (<25 µM); MRSA (<25 µM); *M. luteus* (<25 µM); *Enterococcus faecalis* (VSE) (>25 µg/mL); *E. faecalis* (VanA) (>25 µg/mL); *E. faecalis* (VanB) (>25 µg/mL); *Enterococcus gallinarum* (VanC) (>25 µg/mL)
**5N**	Formoxanthone-C 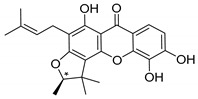	*B. Substilis* (4.6 µM); *S. aureus* (2.3 µM); *Streptococcus faecalis* (18.7 µM); *Salmonella Typhi* (4.6 µM)

MIC: Minimum inhibitory concentration; MRSA: Methicillin-resistant *S. aureus*; MSSA: Methicillin-sensitive *S. aureus*; * Stereogenic center.

**Table 2:** Antimicrobial activity of natural CDXs with pyran groups.

No.	Name/Structure	Antimicrobial Activity (MIC)
**6N**	Mangostanol 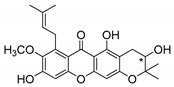	*M. tuberculosis* H_37_Ra (200 µg/mL)
**7N**	Tovophyllin-B 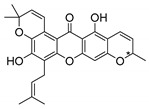	*M. tuberculosis* H_37_Ra (25 µM)
**8N**	Calozeyloxanthone 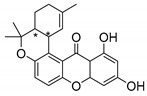	17 MRSA strains and 25 MSSA strains (range 4.1–8.1 µg/mL)
**9N**	Smeathxanthone B 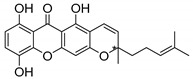	*Escherichia coli* (625 µg/mL), *Klebsiella pneumoniae* (625 µg/mL), *Proteus vulgaris* (312.5 µg/mL), *S. typhi* (625 µg/mL), *S. faecalis* (625 µg/mL), *C. albicans* (312.5 µg/mL), *C. krusei* (312.25 µg/mL)
**10N**	Artoindonesianin-C 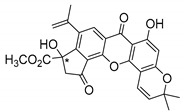	*M. tuberculosis* H_37_Ra (12.5 µM)
**11N**	Cowagarcinone-D 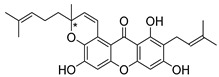	MRSA-SK1 (128 µg/mL); *S. aureus* (inactive); *E. coli* (128 µg/mL); *Salmonella typhimurium* (128 µg/mL)

MIC: Minimum inhibitory concentration; MRSA: Methicillin-resistant *S. aureus*; MSSA: Methicillin-sensitive *S. aureus*; * Stereogenic center.

**Table 3:** Antimicrobial activity of natural CDXs with hydroxy side chains.

No.	Name/Structure	Antimicrobial Activity (MIC)
**12N**	Fuscaxanthone I 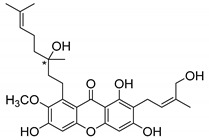	*Helicobacter pylori* ATCC 43504 (30.5 µM); *H. pylori* DMST 20165 (15.2 µM); *H. pylori* HP40 (122.0 µM)
**13N**	Caledol 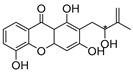	*A. fumigatus* (32 µM); *C. albicans* (inactive)
**14N**	Dicaledol 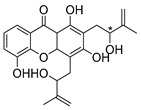	*A. fumigatus* (1 µM); *C. albicans* (inactive)
**15N**	Mangostenol 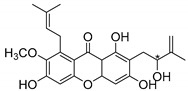	*M. tuberculosis* H_37_Ra (100 µM)

MIC: Minimum inhibitory concentration; * Stereogenic center.

**Table 4:** Antimicrobial activity of natural caged xanthones: scortechinones and prenylated.

No.	Name/Structure	${\left[\alpha \right]}_{D}^{29}(c)$ ^a^	Antimicrobial Activity (MIC)
**16N**	Scortechinone A 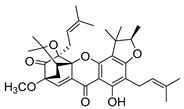	+18° (0.028)	*S. aureus* (128 µg/mL); *S aureus* SK1 (128 µg/mL); MRSA (128 µg/mL)
**17N**	Scortechinone B 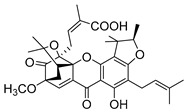	−105° (0.095)	*S. aureus* (8 µg/mL); *S aureus* SK1 (2 µg/mL); MRSA (2 µg/mL)
**18N**	Scortechinone C 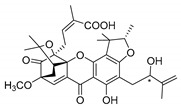	−107° (0.014)	*S. aureus* (32 µg/mL); *S aureus* SK1 (32 µg/mL); MRSA (32 µg/mL)
**19N**	Scortechinone D 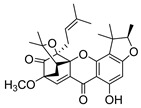	+222° (0.018)	*S. aureus* (>256 µg/mL); *S aureus* SK1 (>256 µg/mL)
**20N**	Scortechinone E 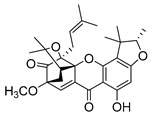	−240° (0.025)	*S. aureus* (>256 µg/mL); *S aureus* SK1 (>256 µg/mL)
**21N**	Scortechinone F 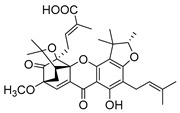	−333° (0.015)	*S. aureus* (16 µg/mL); *S aureus* SK1 (4 µg/mL)
**22N**	Scortechinone G 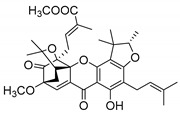	−95° (0.021)	*S. aureus* (>64 µg/mL); *S aureus* SK1 (>64 µg/mL)
**23N**	Scortechinone H 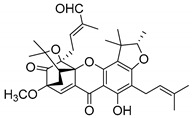	−120° (0.025)	*S. aureus* (>64 µg/mL); *S aureus* SK1 (4 µg/mL)
**24N**	Scortechinone I 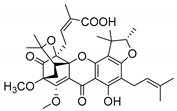	+43° (0.023)	*S. aureus* (8 µg/mL); *S aureus* SK1 (8 µg/mL)
**25N**	Scortechinone J 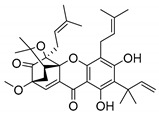	−200° (0.015)	*S. aureus* (32 µg/mL); *S aureus* SK1 (8 µg/mL)
**26N**	Scortechinone K 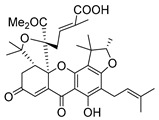	+48° (0.021)	*S. aureus* (128 µg/mL); *S aureus* SK1 (128 µg/mL)
**27N**	Scortechinone L 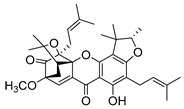	−176° (0.017)	*S. aureus* (>64 µg/mL); *S aureus* SK1 (>64 µg/mL)
**28N**	Scortechinone M 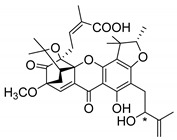	−353° (0.017)	*S. aureus* (32 µg/mL); *S aureus* SK1 (32 µg/mL)
**29N**	Scortechinone N 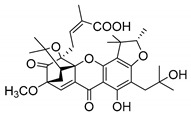	−263° (0.019)	*S. aureus* (32 µg/mL); *S aureus* SK1 (32 µg/mL)
**30N**	Scortechinone O 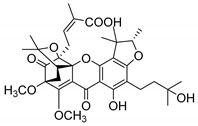	+77° (0.013)	*S. aureus* (>128 µg/mL); *S aureus* SK1 (>128 µg/mL)
**31N**	Scortechinone P 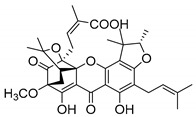	+83° (0.012)	*S. aureus* (32 µg/mL); *S aureus* SK1 (16 µg/mL)
**32N**	2-isoprenylforbesione 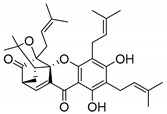		MRSA USA300 SF8300 (7.20 mm ^b^; >400 µM); MSSA ATCC 25923 (7.56 mm ^b^; 400 µM)
**33N**	Deoxygamboginin 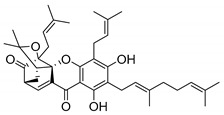		MRSA USA300 SF8300 (6 mm ^b^); MSSA ATCC 25923 (6 mm ^b^)
**34N**	Hanburin 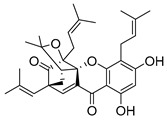		MRSA USA300 SF8300 (6 mm ^b^); MSSA ATCC 25923 (6 mm ^b^)
**35N**	Forbesione 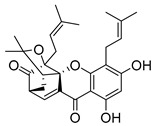		MRSA USA300 SF8300 (7.97 mm ^b^; >400 µM); MSSA ATCC 25923 (7.86 mm ^b^, 200 µM)
**36N**	Dihydroisomorellin 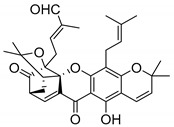		MRSA USA300 SF8300 (6 mm ^b^); MSSA ATCC 25923 (6 mm ^b^)

MIC: Minimum inhibitory concentration; MRSA: Methicillin-resistant *S. aureus*; MSSA: Methicillin-sensitive *S. aureus*; ^a^ Specific rotation measured in methanol; ^b^ The antimicrobial studies were determined using the disc diffusion method, where the inhibitory growth zones inhibition caused by the tested compounds is expressed in millimeters.

**Table 5:** Antimicrobial activity of natural caged xanthones with pyran group.

No.	Name/Structure	${\left[\alpha \right]}_{D}^{28}(c)$ ^a^	Antimicrobial Activity (MIC or Zone of Growth)
**37N**	Hanburinone 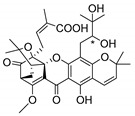	−62° (0.09)	MRSA (200 µM)
**38N**	Isomoreollin B 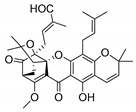	−44° (0.11)	MRSA (200 µM)
**39N**	Morellin 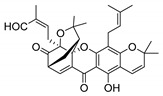	−600° (0.04)	MRSA (200 µM)
**40N**	Moreollic acid 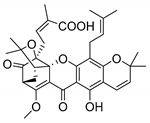	−39° (0.22)	MRSA (25 µM)
**41N**	Morellic acid 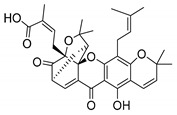	−541° (0.19)	MRSA (25 µM); MRSA USA300 SF8300 (19.52 mm^b^; 12.5 µM); MSSA ATCC 25923 (19.23 mm ^b^; 12.5 µM)
**42N**	Deoxymorellin 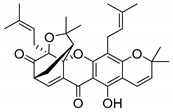		MRSA USA300 SF8300 (6 mm ^b^); MSSA ATCC 25923 (6 mm ^b^)
**43N**	Isomorellinol 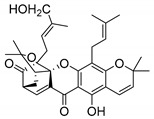		MRSA USA300 SF8300 (8.57 mm ^b^); MSSA ATCC 25923 (7.75 mm ^b^)
**44N**	Gambogic acid 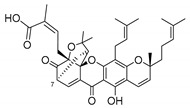	${\left[\alpha \right]}_{D}^{20}$(0.17) = −714.1°	MRSA USA300 SF8300 (17.29 mm ^b^; 25 µM); MSSA ATCC 25923 (16.59 mm ^b^; 12.5 µM)

MIC: Minimum inhibitory concentration; MRSA: Methicillin-resistant *S. aureus*; MSSA: Methicillin-sensitive *S. aureus*; ^a^ Specific rotation measured in CHCl_3_; ^b^ The antimicrobial studies were determined using the disc diffusion method, where the inhibitory growth zones’ inhibition caused by the tested compounds is expressed in millimeters.

**Table 6:** Antimicrobial activity of other natural CDXs.

No.	Name/Structure	Antimicrobial Activity (MIC or Zone of Growth)
**45N**	Kielcorin 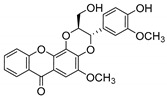 ${\left[\alpha \right]}_{D}^{25}$ = −70.0° (0.01) ^a^	*S. aureus*-1199B (>512 mg/L); MRSA XU212 (>512 mg/L); *S. aureus* ATCC 25923 (>512 mg/L); MRSA RN4220 (>512 mg/L); EMRSA-15 (>512 mg/L); EMRSA-16 (>512 mg/L)
**46N**	Mangiferin 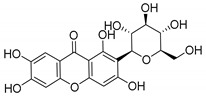	*Bacillus pumilus* (18 mm ^b^); *B. cereus* (15 mm ^b^); *Salmonella enterica serotype Virchow* (22 mm ^b^); *Pseudomonas aeruginosa* (0 mm ^b^); *Aspergillus flavus* (0 mm ^b^; 12 mm ^c^); *Thermoascus aurantiacus* (0 mm ^b^; 18 mm ^c^); *B. cereus* (40 µg/mL); *Mariniluteicoccus flavus* (40 µg/mL); *Listeria monocytogenes* (40 µg/mL); *E. coli* (40 µg/mL); *Enterobacter cloacae* (40 µg/mL); *P. aeruginosa* (40 µg/mL); *S. typhimurium* (40 µg/mL); *Penicillium funiculosum* (40 µg/mL); *Penicillium ochrochloron* (40 µg/mL); *Trichoderma viride* (40 µg/mL); *A. fumigatos* (20 µg/mL); *A. niger* (40 µg/mL); *A. flavus* (40 µg/mL); *A. versicolor* (20 µg/mL); *C. albicans* (40 µg/mL)
**47N**	Buanmycin 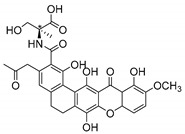 ${\left[\alpha \right]}_{D}^{25}$ = +72.0° (0.5) ^a^	*S. aureus* (10.5 µM); *B. subtilis* (0.7 µM); *Kocuria rhizophila* (10.5 µM)
**48N**	Microluside-A 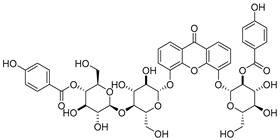	*E. faecalis* JH212 (10 µM); *S. aureus* NCTC 8325 (13 µM)
**49N**	Garmoxanthone 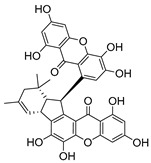	2strains of MRSA (3.9 µg/mL); 2 strains of *Vibrio vulnificus* (15.6 µg/mL); *Vibrio rotiferianus* (15.6 µg/mL); *Vibrio campbellii* (31.2 µg/mL)

^a^ Specific rotation measured in methanol; ^b^ The antimicrobial studies were performed by disc diffusion method, where the inhibitory growth zones inhibition caused by the tested compounds in 15% concentration and ^c^ compounds at 30% concentration are expressed in millimeters.

**Table 7:** Antimicrobial activity of muchimangins analogues.

No.	Structure	${\left[\alpha \right]}_{D}^{24}(c)$ ^a^	Antimicrobial Activity (MIC)
**1S**	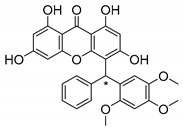	±	*S. aureus* (10.0 µM); *B. subtilis* (50.0 µM)
		+2.5 (0.02)	*S. aureus* (10.0 µM); *B. subtilis* (50.0 µM)
		−28.0 (0.02)	*S. aureus* (12.5 µM); *B. subtilis* (100.0 µM)
**2S**	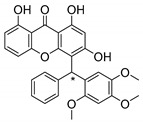	±	*S. aureus* (10.0 µM); *B. subtilis* (12.5 µM)
		+	*S. aureus* (10.0 µM); *B. subtilis* (10.0 µM)
		-	*S. aureus* (10.0µM); *B. subtilis* (12.5 µM)
**3S**	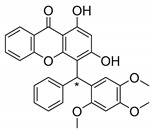	±	*S. aureus* (25.0 µM); *B. subtilis* (>100.0 µM)
		+	*S. aureus* (10.0 µM); *B. subtilis* (>100.0 µM)
		-	*S. aureus* (50.0 µM); *B. subtilis* (>100.0 µM)
**4S**	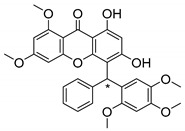	±	*S. aureus* (>100 µM); *B. subtilis* (>100.0 µM)
**5S**	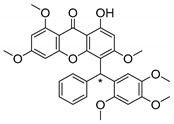	±	*S. aureus* (>100 µM); *B. subtilis* (>100.0 µM)

MIC: Minimum inhibitory concentration; ^a^ Specific rotation measured in CHCl_3_; * Stereogenic center; Enantioselectivity is represented by: “±” racemate; “-“ levorotatory; “+” dextrorotatory.

**Table 8:**
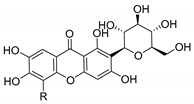
Antimicrobial activity of mangiferin analogues.

No.	R	Antimicrobial Activity (Inhibitory Growth Zones)
**45N**	H (Mangiferin)	*B. pumilus* (18 mm); *B. cereus* (15 mm); *S. virchow* (22 mm); *P. aeruginosa* (0 mm); *A. flavus* (0 mm; 12 mm *); *T. aurantiacus* (0 mm; 18 mm *)
**6S**	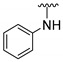	*B. pumilus* (16 mm); *B. cereus* (12 mm); *S. virchow* (19 mm); *P. aeruginosa* (0 mm; 10 mm *); *A. flavus* (0 mm; 11 mm *); *T. aurantiacus* (0 mm; 14 mm *)
**7S**	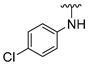	*B. pumilus* (15 mm); *B. cereus* (12 mm); *S. virchow* (20 mm); *P. aeruginosa* (0 mm; 8 mm *); *A. flavus* (0 mm; 11 mm *); *T. aurantiacus* (0 mm; 13 mm *)
**8S**	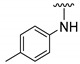	*B. pumilus* (17 mm); *B. cereus* (15 mm); *S. virchow* (20 mm); *P. aeruginosa* (0 mm; 10 mm *); *A. flavus* (0 mm; 14 mm *); *T. aurantiacus* (0 mm; 15 mm *)
**9S**	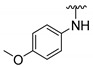	*B. pumilus* (18 mm); *B. cereus* (14 mm); *S. virchow* (20 mm); *P. aeruginosa* (0 mm; 9 mm *); *A. flavus* (0 mm; 11 mm *); *T. aurantiacus* (0 mm; 16 mm *)
**10S**	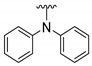	*B. pumilus* (17 mm); *B. cereus* (14 mm); *S. virchow* (19 mm); *P. aeruginosa* (0 mm; 9 mm *); *A. flavus* (0 mm; 12 mm *); *T. aurantiacus* (0 mm; 14 mm *)
**11S**	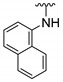	*B. pumilus* (18 mm); *B. cereus* (13 mm); *S. virchow* (18 mm); *P. aeruginosa* (0 mm; 10 mm *); *A. flavus* (0 mm; 11 mm *); *T. aurantiacus* (0 mm; 15 mm *)

The antimicrobial studies were determined using a disc diffusion method, where the inhibitory growth zones inhibition caused by the tested compounds in 15% concentration is expressed in millimeters (mm); * compounds at 15% concentration (with microbial activity) and at 30%.

**Table 9:**
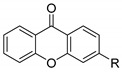
Antimicrobial activity of amino acid xanthone derivatives.

No.	Structure/R	Antimicrobial Activity (Inhibitory Growth Zones/MIC)
**12S**	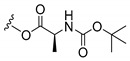	*S. aureus* (6 mm-25 µg/mL); *B. substilis* (8 mm-25 µg/mL); *E. coli* (17 mm-25 µg/mL); *K. pneumonia* (6 mm-25 µg/mL)
**13S**	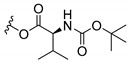	*S. aureus* (10 mm-25µg/mL); *B. substilis* (7 mm-25 µg/mL); *E. coli* (8 mm-25 µg/mL); *K. pneumonia* (5 mm-25 µg/mL)
**14S**	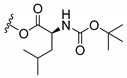	*S. aureus* (7 mm-25 µg/mL); *B. substilis* (11 mm-25 µg/mL); *E. coli* (4 mm-25 µg/mL); *K. pneumonia* (8 mm-25 µg/mL)
**15S**	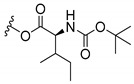	*S. aureus* (4 mm-25 µg/mL); *B. substilis* (7 mm-25 µg/mL); *E. coli* (8 mm-25 µg/mL); *K. pneumonia* (7 mm-25 µg/mL)
**16S**	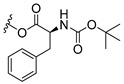	*S. aureus* (18 mm-25 µg/mL); *B. substilis* (17 mm-25 µg/mL); *E. coli* (16 mm-25 µg/mL); *K. pneumonia* (20 mm-25 µg/mL)
**17S**	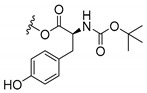	*S. aureus* (20 mm-25 µg/mL); *B. substilis* (20 mm-25 µg/mL); *E. coli* (20 mm-25 µg/mL); *K. pneumonia* (18 mm-25 µg/mL)
**18S**	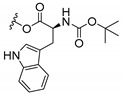	*S. aureus* (22 mm-25 µg/mL); *B. substilis* (23 mm-25 µg/mL); *E. coli* (24 mm-25 µg/mL); *K. pneumonia* (22 mm-25 µg/mL)
**19S**	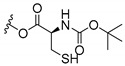	*S. aureus* (20 mm-25 µg/mL); *B. substilis* (20mm-25 µg/mL); *E. coli* (18 mm-25 µg/mL); *K. pneumonia* (18 mm-25 µg/mL)
**20S**	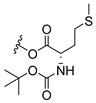	*S. aureus* (15 mm-25 µg/mL); *B. substilis* (13 mm-25 µg/mL); *E. coli* (16 mm-25 µg/mL); *K. pneumonia* (16 mm-25 µg/mL)
**21S**	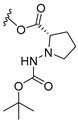	*S. aureus* (17mm-25 µg/mL); *B. substilis* (15mm-25 µg/mL); *E. coli* (17 mm-25 µg/mL); *K. pneumonia* (13 mm-25 µg/mL)
**22S**	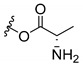	*S. aureus* (9 mm-25 µg/mL); *B. substilis* (11 mm-25 µg/mL); *E. coli* (15 mm-25 µg/mL); *K. pneumonia* (8 mm-25 µg/mL)
**23S**	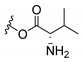	*S. aureus* (14 mm-25 µg/mL); *B. substilis* (10 mm-25 µg/mL); *E. coli* (11 mm-25 µg/mL); *K. pneumonia* (16 mm-25 µg/mL)
**24S**	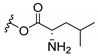	*S. aureus* (9 mm-25 µg/mL); *B. substilis* (13 mm-25 µg/mL); *E. coli* (8 mm-25 µg/mL); *K. pneumonia* (10 mm-25 µg/mL)
**25S**	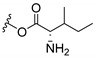	*S. aureus* (7 mm-25 µg/mL); *B. substilis* (11 mm-25 µg/mL); *E. coli* (10 mm-25 µg/mL); *K. pneumonia* (8 mm-25 µg/mL)
**26S**	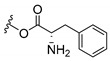	*S. aureus* (22 mm - 25 µg/mL); *B. substilis* (22 mm - 25 µg/mL); *E. coli* (20 mm - 25 µg/mL); *K. pneumonia* (23 mm - 25 µg/mL)
**27S**	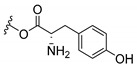	*S. aureus* (23 mm-25 µg/mL); *B. substilis* (23 mm-25 µg/mL); *E. coli* (21 mm-25 µg/mL); *K. pneumonia* (21 mm-25 µg/mL)
**28S**	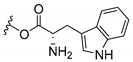	*S. aureus* (24 mm-25 µg/mL); *B. substilis* (26 mm-25 µg/mL); *E. coli* (26 mm-25 µg/mL); *K. pneumonia* (23 mm-25 µg/mL)
**29S**	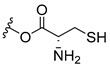	*S. aureus* (20 mm-25 µg/mL); *B. substilis* (19 mm-25 µg/mL); *E. coli* (19 mm-25 µg/mL); *K. pneumonia* (15 mm-25 µg/mL)
**30S**	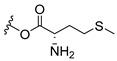	*S. aureus* (17 mm-25 µg/mL); *B. substilis* (15 mm-25 µg/mL); *E. coli* (17 mm-25 µg/mL); *K. pneumonia* (17 mm-25 µg/mL)
**31S**	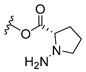	*S. aureus* (20 mm-25 µg/mL); *B. substilis* (18 mm-25 µg/mL); *E. coli* (20 mm-25 µg.mL); *K. pneumonia* (18 mm-25 µg.mL)

The antimicrobial activity was performed in agar well diffusion method, in triplicate, being the results expressed as the mean of the diameter of the inhibition zone in millimeter.

**Table 10:**
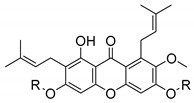
Antimicrobial activity of α-mangostin analogues.

No.	Structure/R	Antimicrobial Activity (MIC)
**32S**	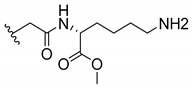	*S. aureaus* (6 µg/mL); MRSA DM21455 (12 µg/mL); MRSA DM09809R (6 µg/mL); *B. cereus* ATCC 11778 (12 µg/mL)
**33S**	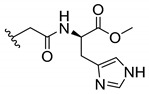	*S. aureaus* (>50 µg/mL); MRSA DM21455 (>50 µg/mL); MRSA DM09809R (>50 µg/mL); *B. cereus* ATCC 11778 (>50 µg/mL); *Mycobacetrium smegmatis* (>24.9 µg/mL); *M. bovis* (>24.9 µg/mL)
**34S**	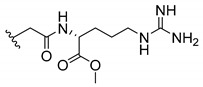	*S. aureaus* DM4001 (2 µg/mL); MRSA DM21455 (3 µg/mL); MRSA DM09809R (3 µg/mL); *B. cereus* ATCC 11778 (2 µg/mL); *M. smegmatis* (>25.1 µg/mL); *M. bovis* (>25.1 µg/mL); MSSA (7 strains) (2–4 µg/mL); VISA (4 µg/mL); MRSA (10 strains) (2-4 µg/mL); EMRSA (3 strains) (2–4 µg/mL); teicoplanin-RI (2 µg/mL); MDR (2 µg/mL); *Staphylococcus epidermidis* (2 strains) (2 µg/mL); VSE (3 strains) (2–4 µg/mL); VRE (5 strains) (2–4 µg/mL); *Streptococcus* (4 strains) (4–8 µg/mL); *Corynebacterium jeikeium* and *L. monocytogenes* (4–8 µg/mL)
**35S**	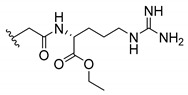	*S. aureaus* (6 µg/mL); MRSA DM21455 (6 µg/mL); MRSA DM09809R (12 µg/mL). *B. cereus* ATCC 11778 (12 µg/mL)
**36S**	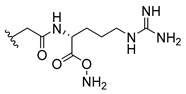	*S. aureaus* DM4001 (6 µg/mL); MRSA DM21455 (1 µg/mL); MRSA DM09809R (6 µg/mL); *B. cereus* ATCC 11778 (6 µg/mL); *M. smegmatis* (>26.1 µg/mL); *M. bovis* (>11.1 µg/mL)
**37S**	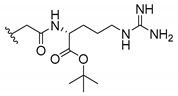	*S. aureaus* (12 µg/mL); MRSA DM21455 (12 µg/mL); MRSA DM09809R (12 µg/mL). *B. cereus* ATCC 11778 (12 µg/mL)
**38S**	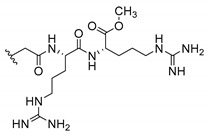	*S. aureaus* (0.5 µg/mL); MRSA DM21455 (2 µg/mL); MRSA DM09809R (3 µg/mL); *B. cereus* ATCC 11778 (3 µg/mL); MSSA (7 strains) 2–4; VISA 2; MRSA (10 strains) (2 µM); EMRSA (3 strains) (2 µM); *teicoplanin*-RI (2 µg/mL); MDR (2 µM); *S. epidermidis* (2 strains) (2 µM); VSE (3 strains) (2–4 µM); VRE (5 strains) (1–2 µM); *Streptococcus* (4 strains) (2–8 µM); *C. jeikeium* and *L. monocytogenes* (2–4 µM)
**39S**	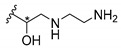	*M. smegmatis* (>19.3 µg/mL); *M. bovis* (>19.3 µg/mL)
**40S**	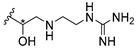	*M. smegmatis* (>21.8 µg/mL); *M. bovis* (>21.8 µg/mL)
**41S**	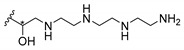	*M. smegmatis* (>24.5 µg/mL); *M. bovis* (>24.5 µg/mL)
**42S**	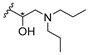	*M. smegmatis* (>21.8 µg/mL); *M. bovis* (>4.6 µg/mL)
**43S**	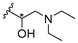	*M. smegmatis* (>4.3 µg/mL); *M. bovis* (>4.3 µg/mL)
**44S**	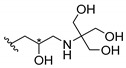	*M. smegmatis* (>19.9 µg/mL); *M. bovis* (>22.9 µg/mL)

MIC: Minimum inhibitory concentration; MRSA: Methicillin-resistant *S. aureus*; MSSA: Methicillin-sensitive *S. aureus*; EMRSA: Epidemic methicillin-resistant *S. aureus*; MDR: Multidrug-resistant bacteria; VRE: Vancomycin-resistant *E.*; VSE: Vancomycin susceptible *E.*; * Stereogenic center.

**Table 11:**
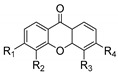
Anti-bacterial activity of xanthone derivatives with 2-hydro-3-amino and piperazine groups.

No.	Structure	Inhibitory Growth Zones [mm] ^a^			
		Other Strains	Clarithromycin Resistant *H. Pylori* Strains	Metronidazole Resistant *H. Pylori* Strains	Double Resistant *H. Pylori* Strains
**45S**	R_1_=R_3_=H; R_2_=Me 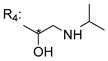	*S. aureaus* ATCC 25923-13; MRSA 14.002-23; *E. coli* ATCC 25922-8	ATCC 700684-36 HP 132/194-40 HP 115/168-40	ATCC 43504-42 HP 125/180-40 HP 139/202-44 HP 143/207-44	HP 126/181-40 HP 106/154-39
**46S**	R_1_=R_3_=H; R_2_=Me 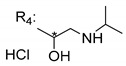	*S. aureaus* ATCC 25923-11; MRSA 14.002-18	ATCC 700684-32 HP 132/194-34 HP 115/168-26	ATCC 43504-35 HP 125/180-36 HP 139/202-46 HP 143/207-29	HP 126/181-40 HP 106/154-33
**47S**	R_1_=R_3_=H; R_2_=Me 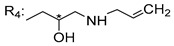	*S. aureaus* ATCC 25923-15; MRSA 14.002-23; *E. coli* ATCC 25922-10	ATCC 700684-34 HP 132/194-42 HP 115/168-46	ATCC 43504-54 HP 125/180-46 HP 139/202-52 HP 143/207-58	HP 126/181-50 HP 106/154-47
**48S**	R_1_=R_2_=R_3_=H 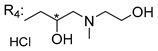		ATCC 700684-28 HP 132/194-30 HP 115/168-30	ATCC 43504-21 HP 125/180-28 HP 139/202-38 HP 143/207-36	HP 126/181-28 HP 106/154-26
**49S**	R_1_=R_2_=R_4_=H 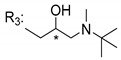	*S. aureaus* ATCC 25923-12; MRSA 14.002-15; *E. coli* ATCC 2592-9	ATCC 700684-35 HP 132/194-42 HP 115/168-38	ATCC 43504-41 HP 125/180-36 HP 139/202-48 HP 143/207-42	HP 126/181-48 HP 106/154-39
**50S**	R_1_=R_2_=R_4_=H 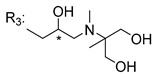		ATCC 700684-24 HP 132/194-22 HP 115/168-17	ATCC 43504-11 HP 125/180-17 HP 139/202-26 HP 143/207-22	HP 126/181-23 HP 106/154-16
**51S**	R_1_=R_2_=R_4_=H 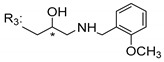	*S. aureaus* ATCC 25923-12; MRSA 14.002-11	ATCC 700684-34 HP 132/194-32 HP 115/168-31	ATCC 43504-36 HP 125/180-40 HP 139/202-40 HP 143/207-32	HP 126/181-34 HP 106/154-31
**52S**	R_1_=R_2_=R_4_=H 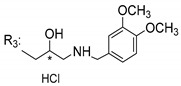		ATCC 700684-20 HP 132/194-16 HP 115/168-25	ATCC 43504-19 HP 125/180-20 HP 139/202-20 HP 143/207-22	HP 126/181-20 HP 106/154-21
**53S**	R_1_=R_2_=R_4_=H 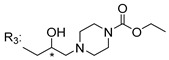		ATCC 700684-27 HP 132/194-29 HP 115/168-34	ATCC 43504-28 HP 125/180-32 HP 139/202-38 HP 143/207-32	HP 126/181-38 HP 106/154-31
**54S**	R_1_=R_2_=R_4_=H 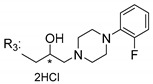		ATCC 700684-25 HP 132/194-30 HP 115/168-33	ATCC 43504-23 HP 125/180-35 HP 139/202-32 HP 143/207-36	HP 126/181-36 HP 106/154-34
**55S**	R_1_=R_2_=R_4_=H 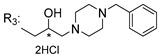	*S. aureaus* ATCC 25923-9; MRSA 14.002-11	ATCC 700684-38 HP 132/194-48 HP 115/168-44	ATCC 43504-39 HP 125/180-50 HP 139/202-54 HP 143/207-50	HP 126/181-56 HP 106/154-45
**56S**	R_1_=Cl; R_2_=R_4_=H 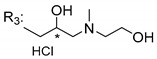	*S. aureaus* ATCC 25923-10; MRSA 14.002-16	ATCC 700684-34 HP 132/194-40 HP 115/168-40	ATCC 43504-37 HP 125/180-40 HP 139/202-40 HP 143/207-48	HP 126/181-45 HP 106/154-40
**57S**	R_1_=Cl; R_2_=R_4_=H 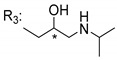	*S. aureaus* ATCC 25923-12; MRSA 14.002-13	ATCC 700684-26 HP 132/194-22 HP 115/168-25	ATCC 43504-25 HP 125/180-32 HP 139/202-28 HP 143/207-23	HP 126/181-25 HP 106/154 -21
**58S**	R_1_=Cl; R_2_=R_4_=H 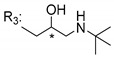	*S. aureaus* ATCC 25923-16; MRSA 14.002-16; *E. coli* ATCC 25922-9	ATCC 700684-35 HP 132/194-44 HP 115/168-46	ATCC 43504-50 HP 125/180-42 HP 139/202-40 HP 143/207-50	HP 126/181-26 HP 106/154-30
**59S**	R_1_=Cl; R_2_=R_4_=H 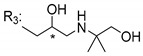	*S. aureaus* ATCC 25923-17; MRSA 14.002-15; *E. coli* ATCC 25922-9	ATCC 700684-34 HP 132/194-36 HP 115/168-38	ATCC 43504-28 HP 125/180-32 HP 139/202-32 HP 143/207-33	HP 126/181-41 HP 106/154-35
**60S**	R_1_=Cl; R_2_=R_4_=H 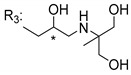	*S.aureaus* ATCC 25923-12; MRSA 14.002-15	ATCC 700684-23 HP 132/194-19 HP 115/168-24	ATCC 43504-15 HP 125/180-18 HP 139/202-31 HP 143/207-26	HP 126/181-20 HP 106/154-19
**61S**	R_1_=Cl; R_2_=R_4_=H 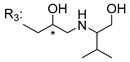	*S. aureaus* ATCC 259-16; MRSA 14.002-16	ATCC 700684-30 HP 132/194-32 HP 115/168-28	ATCC 43504-24 HP 125/180-28 HP 139/202-36 HP 143/207-40	HP 126/181-26 HP 106/154-26
**62S**	R_1_=Cl; R_2_=R_4_=H 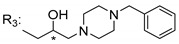		ATCC 700684-22 HP 132/194-24 HP 115/168-27	ATCC 43504-24 HP 125/180-27 HP 139/202-26 HP 143/207-24	HP 126/181-25 HP 106/154-24
**63S**	R_1_=Cl; R_2_=R_4_=H 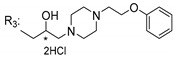	*S. aureaus* ATCC 25923-9; MRSA 14.002-9	ATCC 700684-19 HP 132/194-22 HP 115/168-25	ATCC 43504-22 HP 125/180-29 HP 139/202-30 HP 143/207-25	HP 126/181-27 HP 106/154-25

^a^ The antimicrobial studies were determined using a disc diffusion method, where values correspond to the means of the zones of growth inhibition caused by the tested compounds in 1% concentration in millimeters [110]; * Stereogenic center.

**Table 12:** Antimicrobial activity of derivatives of caged xanthones.

No.	Structure	Antimicrobial Activity (MIC or Inhibitory Growth Zones *)
**64S**	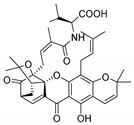	MRSA USA300 SF8300 (22.24 mm; 25 µM); MSSA ATCC 25923 (19.99 mm; 12.5 µM)
**65S**	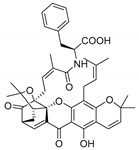	MRSA USA300 SF8300 (16.27 mm; 25 µM); MSSA ATCC 25923 (17.07 mm; 12.5 µM)
**66S**	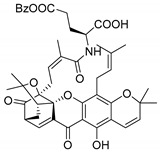	MRSA USA300 SF8300 (6.27 mm); MSSA ATCC 25923 (6.53 mm)
**67S**	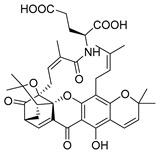	MRSA USA300 SF8300 (6 mm); MSSA ATCC 25923 (6 mm)
**68S**	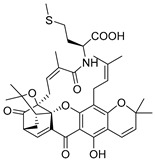	MRSA USA300 SF8300 (9.53 mm); MSSA ATCC 25923 (7.09 mm)
**69S**	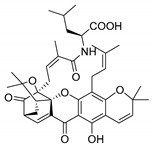	MRSA USA300 SF8300 (18.34 mm; 25 µM); MSSA ATCC 25923 (16.52 mm; 25 µM)
**70S**	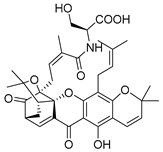	MRSA USA300 SF8300 (6.28 mm); MSSA ATCC 25923 (6.09 mm)
**71S**	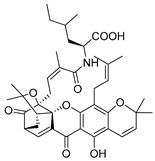	MRSA USA300 SF8300 (19.35 mm; 25 µM); MSSA ATCC 25923 (15.91 mm; 25 µM)
**72S**	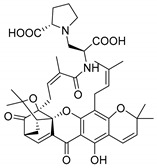	MRSA USA300 SF8300 (15.91 mm; 100 µM); MSSA ATCC 25923 (13.08 mm; 50 µM)
**73S**	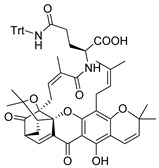	MRSA USA300 SF8300 (8.21 mm); MSSA ATCC 25923 (6.89 mm)
**74S**	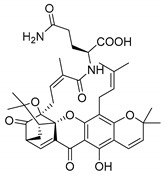	MRSA USA300 SF8300 (9.09 mm); MSSA ATCC 25923 (7.50 mm)
**75S**	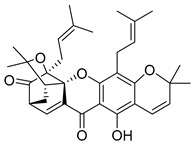	MRSA USA300 SF8300 (6 mm; 25 µM); MSSA ATCC 25923 (6 mm; 12.5 µM)

* The antimicrobial studies were determined by disc diffusion method; MIC: Minimum inhibitory concentration; MRSA: Methicillin-resistant *S. aureus*; MSSA: Methicillin-sensitive *S. aureus*.

**Table 13:**
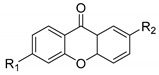
Antimicrobial activity of xanthone derivatives of C-2-substituted.

No.	Structure	Antimicrobial Activity (Inhibitory Growth Zones or MIC)
**76S**	R_1_=H 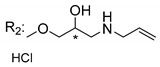	^a^*C. lusitaniae* (8 mm); *T. mentagrophytes* (18 mm); *S. aureus* (9 mm); *E.faecalis* (9 mm)
**77S**	R_1_=Cl 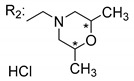	^a^*T. mentagrophytes* (12 mm)
**78S**	R_1_=H 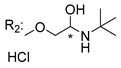	*M. tuberculosis* H_37_Rv (>2.5% with 35% inhibition)
**79S**	R_1_=H 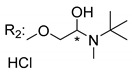	*M. tuberculosis* H_37_Rv (>2.5% with 32% inhibition)
**80S**	R_1_=H 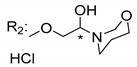	*M. tuberculosis* H_37_Rv (>2.5% with 35% inhibition)
**81S**	R_1_=H 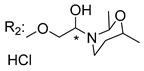	*M. tuberculosis* H_37_Rv (>2.5% with 34% inhibition)
**82S**	R_1_=H 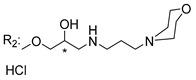	*M. tuberculosis* H_37_Rv (>2.5% with 63% inhibition)
**83S**	R_1_=H 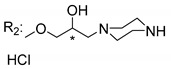	*M. tuberculosis* H_37_Rv (>2.5% with 3% inhibition)
**84S**	R_1_=H 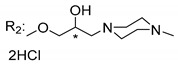	*M. tuberculosis* H_37_Rv (>2.5% with 25% inhibition)
**85S**	R_1_=H 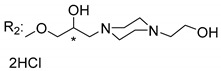	*M. tuberculosis* H_37_Rv (>2.5% with 14% inhibition)
**86S**	R_1_=H 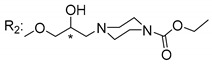	*M. tuberculosis* H_37_Rv (<2.5% with 94% inhibition)
**87S**	R_1_=H 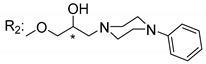	*M. tuberculosis* H_37_Rv (>2.5% with 24% inhibition)
**88S**	R_1_=H 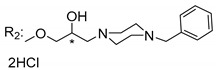	*M. tuberculosis* H_37_Rv (>2.5% with 59% inhibition)

MIC: Minimum inhibitory concentration; ^a^ The antimicrobial studies were determined using a disc diffusion method, where the inhibitory growth zones showed inhibition at 1% concentration against representative strains of microorganisms *C. albicans*, *C. glabrata*, *C. krusei*, *C. lusitaniae*, *C. neoformans*, *A. fumigatus*, *T. mentagrophytes*, *S. aureus*, *E. faecalis*, *E. coli*, *K. pneumonia*, and *P. aeruginosa*; only the strains with activity were expressed; * Stereogenic center.
